# Legislation

**Published:** 1901-06

**Authors:** W. W. Griest

**Affiliations:** Secretary of the Commonwealth


### LEGISLATION.

The following act has been passed by the Pennsylvania State Legislature and was approved by the Governor May 28, 1901:

To provide for the prevention of the spread of disease from the carcasses of animals that die of dangerous or virulent disease, or are killed while afflicted with such disease; to provide for the safe disposal or destruction of such carcasses; to authorize the State Live-stock Sanitary Board to make regulations for the enforcement of this act, and to provide penalties for the violations of this act and of the regulations that may be made under it by the State Live-stock Sanitary Board.

*Carcasses of Diseased Domestic Animals.* Section 1. Be it enacted, etc., That when any domestic animal may die of, or be killed while afflicted with, an infectious, contagious, germ or parasitic disease, adjudged by the State Live-stock Sanitary Board to be of a dangerous or virulent character, and in particular when any domestic animal may die or be killed while it is afflicted with any one of the diseases known as anthrax, black quarter, hog-cholera, swine-plague, rabies, or glanders, the owner or owners of such animal shall at once destroy or dispose of the carcass of such animal by one of the methods herein provided.

*Destruction or Disposal of Carcasses—Methods to be Used.* Section 2. The methods of destruction or disposal shall be of a kind that will completely destroy or securely sequester the poison, germ, parasite or infective agent of the disease with which the animal was afflicted at the time of death. The following methods of destruction or disposal shall be allowed: *One.* Complete burning or cremation of the carcass, and of all its parts and products. *Two.* Boiling the carcass and all of its parts and products in water, or heating the same with steam, at the temperature of boiling water, for at least two hours. *Three.* Burying the carcass and all of its parts and products in a place that is not subject to overflow from ponds or streams, that is distant not less than one hundred feet from any water-course, well, spring, public highway or building used as a house or stable, and in the following manner, to wit: The grave shall be of such a depth that when the carcass and the parts and products thereof are placed in it, and the grave is filled with earth and the top is smoothed to the level of the surrounding surface, the uttermost part of the carcass and of its parts and products shall be completely covered; and, further, the grave shall be so protected that the carcass cannot be dug out or exposed by dogs or other animals. Before the carcass and its parts and products are covered with earth they shall be covered with lime, to a depth of not less than three inches. Any other method of destroying or disposing of carcasses, and of the parts and products of carcass, may be practised that is specifically approved by the State Live-stock Sanitary Board.

*Neglect to Destroy or Dispose of Carcasses—Misdemeanor—Fine.* Section 3. If any person owning an animal that dies while it is afflicted with anthrax, black quarter, hog-cholera, swine-plague, rabies, or glanders, or any other infectious, contagious, germ or parasitic disease, that is adjudged by the State Live-stock Sanitary Board to be of a dangerous or virulent character, shall, after notification by anyone, neglect within twenty-four hours to destroy or dispose of the carcass and its parts and products in accordance with the provisions of section two of this act, the said person shall be deemed guilty of a misdemeanor, and upon conviction thereof shall be punished by a fine of not less than ten dollars nor more than one hundred dollars, at the discretion of the court.

*Notice to Board of Health—To Township Auditor—Duty of Agent of State Live-stock Sanitary Board.* Section 4. When the carcass and products of any animal that died while afflicted with any of the diseases specified in section one of this act, or of any infectious, contagious, germ or parasitic disease, adjudged by the State Live-stock Sanitary Board to be of a dangerous or virulent character, is not disposed of or destroyed in one of the ways set forth in section two of this act, and this fact shall be brought to the attention of an agent of the State Live-stock Sanitary Board, the board of health of the township, borough or city in which the death occurs or in which the carcass of the animal may be; or when this fact shall be brought to the attention of any member of such board of health; or in the event that there is no board of health having jurisdiction, when any township auditor, of a township in which such a carcass may be, is notified of the fact, it shall be the duty of the said agent of the State Live-stock Sanitary Board, or member of a board of health, or said health board, or said township auditor, to at once cause the carcass and its parts and products to be disposed of or destroyed in accordance with the methods prescribed in section two of this act.

*Itemized Bill of Expenses—Proviso.* The disposal or destruction of the carcass shall be carried out in a way that is as economical as is compatible with efficiency and safety, and a full itemized bill of the expense incurred shall be drawn up by the agent of the State Live-stock Sanitary Board, the board of health, or the board of township auditors, and forwarded as a voucher to the State Live-stock Sanitary Board. If the voucher is approved by said board, it shall be paid in the same manner as other expenses of said board are paid: Provided, however, That no charge shall be paid of more than ten dollars for the destruction of a single carcass of a horse, mule, cow, bull, or ox; nor more than three dollars for the destruction of a single carcass of a colt, sheep, hog, or dog.

*Cost of Destruction of Carcass to be a Lien on Property of Owners.* Section 5. The cost of the destruction of the carcass or carcasses, as hereinbefore provided, shall constitute a lien on the property of the owner or owners of the animals at the time of their death; and it shall be the duty of the State Live-stock Sanitary Board to attempt to recover, and if possible to recover, by due process of law, from said owner or owners the amounts expended by it for disposing of or destroying the carcasses of their animals, in the enforcement of this act.

Approved the 2d day of May, A.D. 1901.

WILLIAM A. STONE.

The foregoing is a true and correct copy of the Act of the General Assembly No. 88. W. W. GRIEST,

Secretary of the Commonwealth.

